# Retraction Note: Determinants of viral load suppression failure among HIV adults on ARV attending health care facilities: a retrospective study in Tanga region, Tanzania

**DOI:** 10.1186/s12879-024-09998-3

**Published:** 2024-09-30

**Authors:** Eric Mboggo, Expeditho Mtisi, Stella E. Mushy, Simon Mkawe, Frida Ngalesoni, Aisa Muya, Edwin Kilimba, Denice Kamugumya, Boniface Silvan Mlay

**Affiliations:** 1 AMREF Health Africa, Dar es Salaam, Tanzania; 2 Department of General Studies, Dar Es Salaam Institute of Technology, Dar es Salaam, Tanzania; 3 Department of Community Health Nursing, Muhimbili University of Health Science, Dar es Salaam, Tanzania; 4 Center for Disease Control and Prevention, Dar es salaam, Tanzania; 5 National AIDS Control Program, Ministry of Health, Dodoma, Tanzania

**Retraction Note: BMC Infectious Diseases (2023) 24:312**

10.1186/s12879-023-08604-2

The Editor has retracted this article because it contains material published without permission from the U.S. Centers for Disease Control and Prevention (CDC), the agency that funded the reported research.

Eric Mboggo, Denice Kamugumya, Edwin Kilimba, Simon Mkawe, Aisa Muya, and Frida Ngalesoni agree with this retraction. The remaining authors did not respond to correspondence from the publisher regarding this retraction.

